# Integrating microbial profiling and machine learning for inference of drowning sites: a forensic investigation in the Northwest River

**DOI:** 10.1128/spectrum.01321-24

**Published:** 2024-12-09

**Authors:** Qin Su, Xiaofeng Zhang, Xiaohui Chen, Zhonghao Yu, Weibin Wu, Qingqing Xiang, Chengliang Yang, Jian Zhao, Ling Chen, Quyi Xu, Chao Liu

**Affiliations:** 1 Guangzhou Key Laboratory of Forensic Multi-Omics for Precision Identification, School of Forensic Medicine, Southern Medical University, Guangzhou, Guangdong, China; 2 Guangzhou Forensic Science Institute & Key Laboratory of Forensic Pathology, Ministry of Public Security, Guangzhou, Guangdong, China; 3 National Anti-Drug Laboratory Guangdong Regional Center, Guangzhou, Guangdong, China; University of Minnesota Twin Cities, St. Paul, Minnesota, USA

**Keywords:** submersion time, animal species, cross-species, machine learning, inference of drowning sites

## Abstract

Drowning incidents present significant challenges for forensic investigators in determining the exact site of occurrence. Traditional forensic methods often rely on physical evidence and circumstantial clues, but the emerging field of forensic microbiology offers a promising avenue for enhancing precision and reliability in site inference. Our study investigates the application of microbiome analysis in inferring drowning sites, focusing on microbial diversity in water samples and lung tissues of drowned animals from different sites in the Northwest River. We utilized 16S rDNA sequencing to analyze microbial diversity in water samples and lung tissues, revealing distinct microbial signatures associated with drowning sites. Our findings highlight variations in species richness and diversity across different sampling points, indicating the influence of environmental factors on microbial community structure. Machine learning models trained on microbial data from lung tissues demonstrated high accuracy in predicting drowning sites, with cross-validation accuracy ranging from 83.53% ± 3.99% to 95.07% ± 3.17%. Notably, the Gradient Boosting Machine (GBM) method achieved a classification accuracy of 95.07% ± 3.17% for different sampling points at a submersion time of 1 day. Moreover, our cross-species site inference results revealed that utilizing data from drowned mice to predict the drowning sites of rabbits in location W5 achieved an accuracy of 72.22%. In conclusion, our study underscores the potential of microbiome analysis in forensic investigations of drowning incidents. By integrating microbial data with traditional forensic techniques, there is significant potential to enhance the reliability of scene inferences, thereby making substantial contributions to case investigations and judicial trials.

**IMPORTANCE:**

By employing advanced techniques like microbial profiling and machine learning, the study aims to enhance the accuracy of determining drowning sites, which is crucial for both legal proceedings. By analyzing microbial diversity in water samples and drowned animal lung tissues, the study sheds light on how environmental factors and victim-related variables influence microbial communities. The findings not only advance our understanding of forensic microbiology but also offer practical implications for improving investigative techniques in cases of drowning.

## INTRODUCTION

The role of forensic medicine in the criminal justice system is becoming increasingly crucial, as the intelligence it provides can expedite the resolution of cases (1, 2). Forensic microbiology, as an emerging scientific discipline, shows tremendous potential in aiding criminal investigations (3). Drowning ranks among the top three leading causes of accidental fatalities worldwide, comprising 7% of all injury-related deaths (4). Forensic microbiology not only plays a pivotal role in ascertaining the cause of drowning fatalities but also holds significant importance in pinpointing the exact drowning site (5). Owing to the influence of water currents, the site of body retrieval from water often diverges from the actual site of drowning (6).

Due to the gold standard role of diatoms in distinguishing drowning as a cause of death, many forensic researchers also posit the potential of diatoms in inferring drowning sites (7, 8). Specifically, drowning is primarily determined as the cause of death based on the density and diatom species from multiple organs, whether they belong to the drowning site (9–12). Furthermore, forensic pathology can provide evidence of drowning sites by calculating matches between diatom profiles from tissues and reference water samples (7, 12, 13). However, the identification and classification of diatoms require specialized knowledge and skills, along with substantial human and material resources, making it challenging to conduct widespread surveys of water samples. Therefore, there is a continued need to develop new and reliable methods for inferring drowning sites.

Over the past decade, aquatic bacteria (bacterioplankton) have garnered significant attention from forensic researchers (14–16). These bacteria, which are much smaller (0.2–2μm) and more diverse than diatoms (2 to >500 µm), are more abundant in aquatic environments (15). Utilizing 16S-rDNA microbial profiling allows for high-throughput acquisition of species and community structure distribution information of aquatic bacteria. The integration of machine learning with microbiome research holds tremendous promise in overcoming the challenges of diagnosing drowning sites and is a potentially effective tool in drowning cause of death determination and postmortem interval (PMI) (17).

Our recent studies also demonstrated that by sequencing the 16S-rDNA of lung tissues from drowned rabbits and utilizing genus-level data, we constructed a random forest model (6). This model accurately predicted drowning sites in the test set with 100% accuracy. However, further validation is necessary across multiple geographic locations to assess its robustness and generalizability. In addition, factors such as submersion time and animal species may influence the microbial communities present in the lungs, which could affect the model’s performance. Investigating these variables is essential to refine the model and enhance its accuracy across diverse scenarios. The integration of machine learning with microbial profiling offers a significant advancement in forensic investigations of drowning incidents. To fully realize the potential of this approach, further research exploring the effects of submersion time, species differences, and varying aquatic environments is needed.

## MATERIALS AND METHODS

### Water sample collection

To investigate the distribution of aquatic bacteria in the Northwest River, we selected eight random sampling points (designated as N1, N2, N3, W1, W2, W3, and W4), each spaced 50 km apart as illustrated in Fig. S1. At each location, five 15 mL water samples were collected from depths over 30 cm below the river surface using sterilized plastic bottles on 2 December 2021. The sampling process was conducted within a clearly defined and continuous area measuring 100 cm × 100 cm, located 150 cm from the riverbank. Immediately after collection, the water samples were transported to the forensic laboratory. Samples were filtered through HL-6 multi-unit vacuum filters (0.22 µm pore size) and placed in 50 mL sterile centrifuge tubes for further analysis.

For sample preservation, all collected samples were rapidly frozen in liquid nitrogen and stored in a −80°C freezer. In addition, 50 L of water was collected from each sampling point within the same defined area for subsequent animal experiments, with all water samples utilized within 24 hours.

### Physicochemical analysis of water

Physicochemical factors of the water samples from each collection point were analyzed using specific instruments before animal experiments. The pH values were measured using a Leici PHS-3C pH meter (Leici, China). Total nitrogen content was determined using a Shimadzu UVmini-1240 ultraviolet spectrophotometer (Shimadzu, Japan), while total phosphorus, nitrate, and ammonia nitrogen were measured using a Shimadzu UV-Vis spectrophotometer model UV-1800 (Shimadzu, Japan). Dissolved oxygen was measured using a YSI5000 dissolved oxygen meter (Yellow Springs Instrument Company, USA). Other data were determined using titration methods.

### Animal experiments and tissue sample collection

We obtained 450 eight-week-old male C57BL/6 mice and 12 three-month-old New Zealand female rabbits from the Experimental Animal Center of Southern Medical University. For each water sample retrieved from the sampling points, 54 mice were immersed in the corresponding water sample until they succumbed. In addition, the drowned mice were left in the respective water samples, with 18 mice left for 1 day, 18 for 4 days, and 18 for 7 days before their lungs were extracted. The lungs of drowned mice from different sampling locations were labeled as M-N1, M-N2, M-N3, M-W1, M-W2, M-W3, M-W4, and M-W5. Furthermore, they were categorized into groups based on the submersion time, namely the Day 1 group, the Day 4 group, and the Day 7 group. In addition, 18 mice were euthanized by cervical dislocation in the laboratory, and their lungs were extracted after 1 day. These mice were labeled as the M-BC group. At sampling points N1 and W5, six rabbits were immersed in the corresponding water samples until they succumbed, and their lungs were extracted after 1 day. These rabbits were labeled as the R-N1 and R-W5 groups, respectively. Three random tissue samples were taken from each rabbit’s lung for subsequent experiments. After extraction, the lung samples from mice and rabbits were rapidly frozen in liquid nitrogen and stored in a −80°C freezer. All animal experiments were conducted following the guidelines and regulations approved by the Animal Ethics Committee of Southern Medical University (Approval No. L2020064).

### DNA extraction

To extract DNA from the filtered water samples, we used sterilized scissors to cut the filters into small pieces, which were then mixed with 20 µL of proteinase K and 20 µL of DTT. DNA extraction was performed using the DNeasy PowerSoil Kit (Qiagen, Hilden, Germany). For tissue DNA extraction, 20 g of tissue samples was ground into powder in liquid nitrogen, followed by DNA extraction using the E.Z.N.A. Water DNA Kit (Omega, USA) according to the manufacturer’s instructions.

### PCR amplification and sequencing

The V3-V4 region of the 16S rDNA was amplified using primers 340F and 805R (340F: 5′-CCTACGGGNBGCASCAG-3′, 805R: 5′-GACTACNVGGGTATCTAATCC-3′). The first PCR mixture included 12.5 µL of 2 × KAPA HiFi HotStart Ready Mix buffer (Kapa Biosystems, Wilmington, USA), 2.5 µL of extracted DNA (5 ng/µL), and 5 µL of each primer (1 µM). The amplification process consisted of an initial denaturation-activation step at 95°C for 3 min, followed by 25 cycles of denaturation at 95°C for 30 s, annealing at 55°C for 30 s, and extension at 72°C for 30 s. PCR products were confirmed by 2% agarose gel electrophoresis, purified with magnetic beads, and used as templates for the second PCR. Ultrapure water was used in place of the sample solution throughout DNA extraction, serving as a negative control to rule out false-positive PCR results. The PCR products were then purified using AMPure XT beads (Beckman Coulter Genomics, Danvers, MA, USA) and quantified with a Qubit fluorometer (Invitrogen, USA). For sequencing preparation, the amplicon pools were further evaluated for size and concentration using an Agilent 2100 Bioanalyzer (Agilent, USA) and quantified with the Library Quantification Kit for Illumina (Kapa Biosciences, Woburn, MA, USA). Finally, the samples were sequenced on the Illumina NovaSeq 6000 platform with PE250 sequencing. Relevant 16S rDNA sequencing raw data have been deposited in the Genbank repository under the accession number PRJNA1181121.

### Bioinformatics and statistical analysis

The raw data underwent quality control using the fastp software (version 0.20.01) (18), followed by sequence merging using the FLASH software (version 1.2.72) (19). The resulting sequences were denoised using Deblur (20) and then imported into QIIME2 (version 138) (21). After classification and normalization of 16S rDNA copy numbers using classify-sklearn, the data were merged at different levels. Pairwise comparisons of Observed_OTUs, Chao1, Shannon, and Simpson indices were conducted using the Kruskal-Wallis test. Differences between groups with statistical significance (*P* < 0.05) were indicated by different lowercase letters. A heatmap of relative abundance levels of bacteria was generated using the tool located at https://www.omicstudio.cn/tool/124. The Mantel correlation heatmap was generated using the tool at https://www.omicstudio.cn/tool/109. Machine learning models were built using the H2O package in the R language. H2O is a powerful open-source machine learning framework with Automated Machine Learning (AutoML) capabilities, widely used for analyzing and modeling large-scale data sets. The models were all created using the H2O package’s AutoML, which automatically attempts multiple types of models, adjusts model hyperparameters automatically, and selects the best-performing model on the given data set. For model validation, the data set was randomly divided into five partitions to implement a fivefold cross-validation approach. In each fold, four subsets were used for training, while the remaining subset was reserved for validation. This cross-validation not only improves model reliability but also helps detect overfitting, as consistent performance across folds indicates a model that generalizes well across unseen data. The learning curves generated from these models are instrumental in assessing the model’s performance, particularly by observing error trends as training data size increases. If the error rate stabilizes or decreases with more data, it indicates a well-generalized model, whereas substantial error fluctuations or discrepancies between training and validation errors may suggest overfitting. The SHAP value plots for model interpretation were also generated using the H2O package for post-model evaluations.

## RESULTS

### Diversity analysis of Northwest River water samples

**Fig 1:**
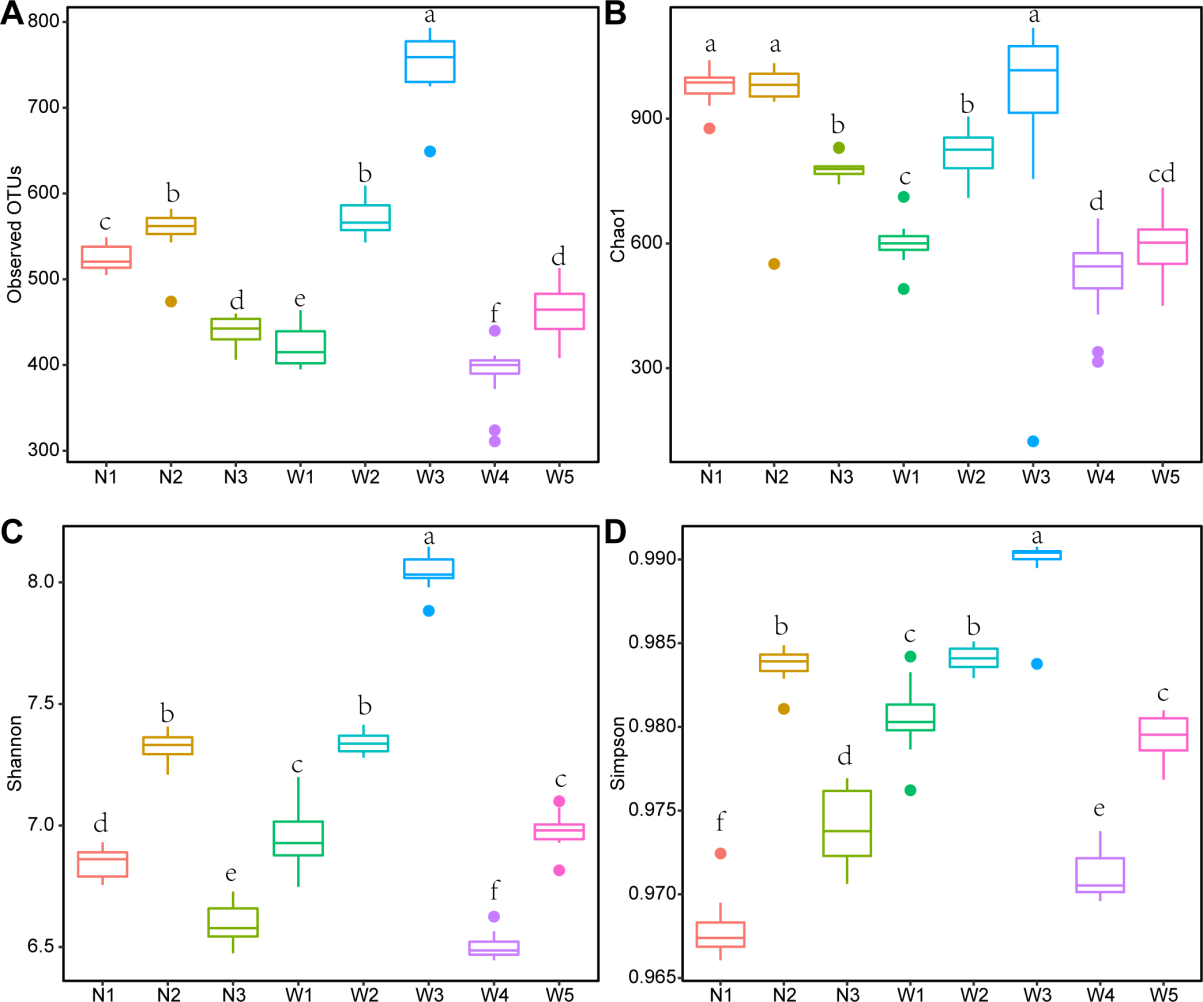
Alpha diversity indices of eight sampling locations (A: Observed OTU numbers; B: Chao1 index; C: Shannon index; D: Simpson index. Different letters indicate statistical differences (*P* < 0.05)).

To analyze the complexity of microbial communities within Northwest River ecosystems, we examined species, abundance, and diversity indices in water samples using 16S rDNA sequencing. The observed number of OTUs and Chao1 index were utilized to represent the richness of species structure within the community, with higher values indicating greater species diversity (22, 23). The highest observed OTU number was at sampling point W3, with a value of 749.55 ± 12.37 (Fig. 1A). Conversely, the lowest observed OTU number was at sampling point W4, at only 388.92 ± 10.61. The Chao1 index was higher at N1, N2, and W3, with values of 976.31 ± 12.31, 950.54 ± 37.35, and 926.85 ± 78.69, respectively (Fig. 1B). By contrast, it was lower at W1, W4, and W5, with values of 600.51 ± 14.76, 519.00 ± 30.90, and 594.04 ± 25.08, respectively. On the other hand, the Shannon index and Simpson index measured species diversity, reflecting the richness and evenness of species within the community (24). Higher index values indicated a more even distribution of species (25). The Shannon index showed the highest diversity at sampling point W3, with a value of 8.04 ± 0.02, followed by N2 and W2, with values of 7.32 ± 0.02 and 7.34 ± 0.01, respectively (Fig. 1C). The lowest Shannon index was observed at sampling point W4, with a value of 6.50 ± 0.01, slightly higher than the value at N3, which was 6.6 ± 0.02. The Simpson index indicated the highest diversity at sampling point W3, with a value of 0.990 ± 0.001, followed by N2 and W2, with values of 0.984 ± 0.001 (Fig. 1D). The lowest Simpson index was observed at sampling point N1, with a value of 0.968 ± 0.001, slightly higher than the value at W4, which was 0.971 ± 0.001. These results provide insights into the microbial diversity of the Northwest River water samples, highlighting variations in species richness and diversity across different sampling points.

In addition, water quality measurements were conducted at each sampling point, encompassing eight parameters including pH, salinity, conductivity, ammonia nitrogen, nitrate, total nitrogen, dissolved oxygen, and total phosphorus, as presented in Table S1. Correlation analysis with alpha diversity data (Fig. S2) revealed highly positive correlations between salinity and conductivity, nitrate and total nitrogen. Strong negative correlations were observed between total phosphorus and pH. Furthermore, pH showed strong positive correlations with salinity and conductivity; dissolved oxygen exhibited strong positive correlations with nitrate and total nitrogen. A significant positive correlation was observed between Chao1 and total phosphorus. Simpson index demonstrated a strong negative correlation with pH and total phosphorus, though without statistical significance. These results indicate interdependencies among water quality indicators, further influencing the Chao1 and Simpson indices of microbial communities, thereby affecting the richness and evenness of species structure within the microbial community.

**Fig 2:**
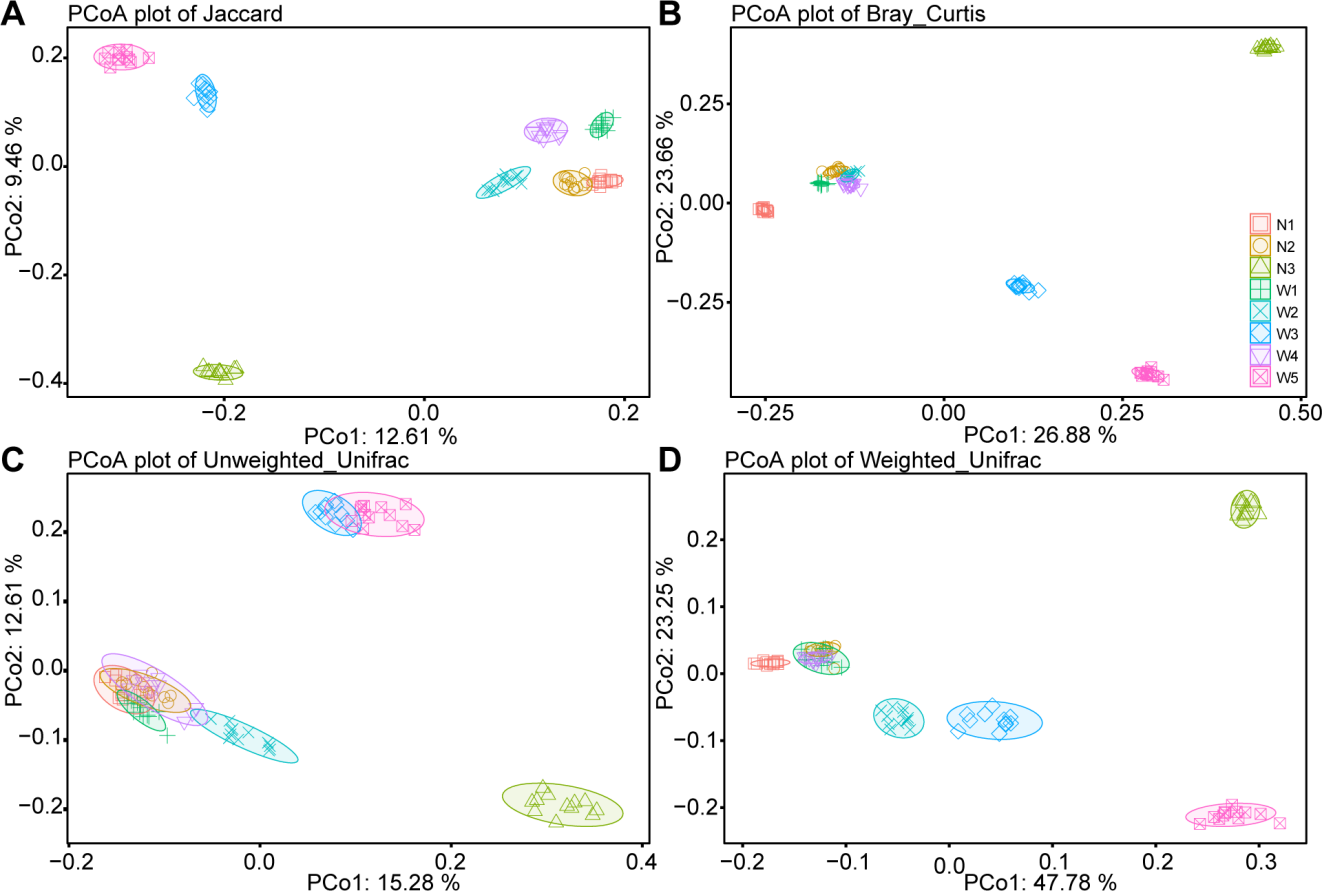
PCoA of Beta diversity of microbial communities in water samples from eight sampling locations. (**A**) PCoA based on Jaccard distance. (**B**) PCoA based on Bray_Curtis distance. (**C**) PCoA based on Unweighted_Unifrac distance. (**D**) PCoA based on Weighted_Unifrac distance.

To further compare the microbial Beta diversity among the eight sampling points in the Northwest River, principal coordinate analysis (PCoA) analysis was conducted based on Jaccard, Bray_Curtis, Unweighted_UniFrac, and Weighted_UniFrac distances (Fig. 2). The results revealed significant differentiation in microbial Beta diversity among the eight sampling points. Among the four distance metrics, sampling point N3 showed the furthest distance from the other seven sampling points, followed by W5 and W3. Distinctions between the eight sampling points were particularly pronounced when considering the abundance of each species (Bray_Curtis and Weighted_UniFrac distances, Fig. 2B and D). However, when considering only bacterial species (Jaccard and Unweighted_UniFrac distances, Fig. 2A and C), the inter-group differences were relatively reduced, especially evident in the overlap between N1, N2, W1, and W4 at Unweighted_UniFrac distances (Fig. 2C).

### Bacterial community structure in Northwest River water samples

**Fig 3:**
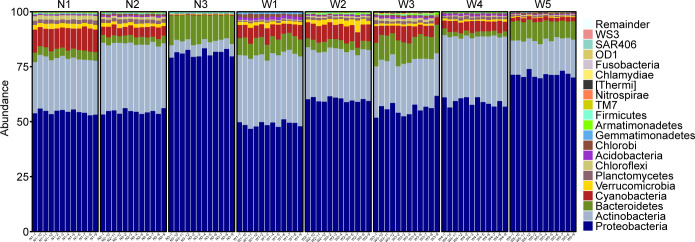
Bar chart of bacterial classification (at phylum level) in microbial communities from eight sampling locations.

Analysis of bacterial community structures in water samples from different sampling points in the Northwest River, at the phylum-level classification, revealed dominant bacterial taxa primarily belonging to the phylum Proteobacteria, accounting for 60.61% ± 1.02% of the 16S rDNA gene sequences (Fig. 3). Among these, the N3 group exhibited the highest proportion at 81.06%, followed by the W5 group (71.31%), while the W1 group had the lowest proportion at 48.83%. The next abundant phyla were Actinobacteria and Bacteroidetes, accounting for 22.79% ± 0.85% and 6.58% ± 0.35% of the 16S rDNA gene sequences, respectively. Actinobacteria showed higher proportions in the W1 and N2 groups, accounting for 31.51% and 30.90%, respectively, while it was lowest in the N3 group at only 5.35%. Bacteroidetes exhibited higher proportions in the N3, W3, and W5 groups, accounting for 11.95%, 10.67%, and 8.46%, respectively. The correlation analysis with water quality indicators, as depicted in Fig. S3, revealed significant positive correlations between Actinobacteria and Bacteroidetes with salinity and conductivity. In addition, Proteobacteria showed strong positive correlations with salinity, conductivity, and dissolved oxygen levels.

### Diversity analysis of mouse drowned lung tissues in Northwest River

**Fig 4:**
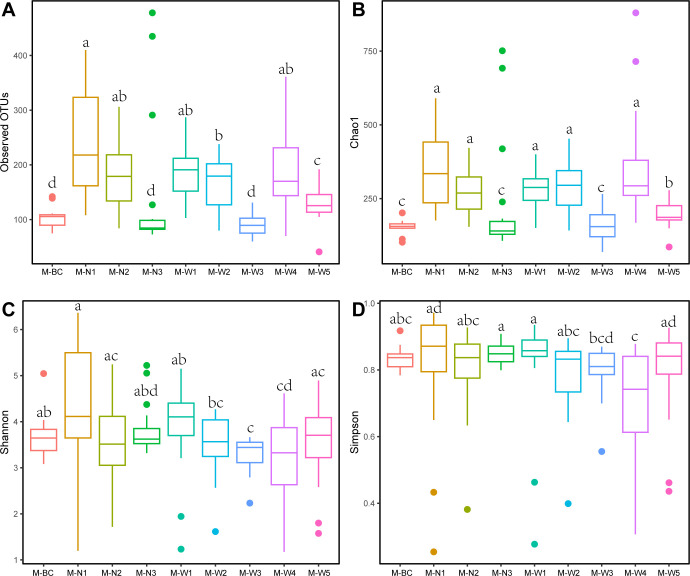
Alpha diversity indices of microbial communities in lung tissues of drowned mice from eight sampling locations (A: Observed OTU numbers; B: Chao1 index; C: Shannon index; D: Simpson index. Different letters indicate statistical differences (*P* < 0.05)).

Analysis of microbial 16S rDNA sequencing results from lung tissues of mice drowned in water samples from different sampling points in the Northwest River, Day 1 groups (Fig. 4), revealed varying levels of microbial diversity. The M-N1 group exhibited the highest number of Observed OTUs, at 241.83 ± 23.32, while the M-BC, M-N3, M-W3, and M-W5 groups showed lower counts, at 104.83 ± 5.68, 139.39 ± 29.62, 92.17 ± 5.37, and 129.17 ± 7.64, respectively (Fig. 4A). Similar to the Observed OTUs index, the Chao1 index showed higher values in the M-W4 and M-N1 groups, at 363.53 ± 43.84 and 347.69 ± 29.92, respectively, while the M-BC, M-N3, M-W3, and M-W5 groups had lower values, at 153.09 ± 7.50, 221.00 ± 46.18, 161.29 ± 13.40, and 195.06 ± 10.47, respectively (Fig. 4B). The results of Shannon and Simpson indices indicated relatively even distributions across the groups, except for the M-W4 group which had the lowest values for both Shannon (3.18 ± 0.21) and Simpson (0.73 ± 0.04) indices (Fig. 4C and D). The association analysis between alpha diversity data and water quality indicators (Fig. S4) revealed significant correlations. Observed OTUs and Chao1 indices showed a highly positive correlation with salinity and conductivity (*P* < 0.05), indicating that higher levels of salinity and conductivity were associated with increased microbial richness. By contrast, a highly negative correlation was observed between Observed OTUs and Chao1 indices with total phosphorus (*P* ≥ 0.05), suggesting that higher levels of total phosphorus were associated with reduced microbial richness.

**Fig 5:**
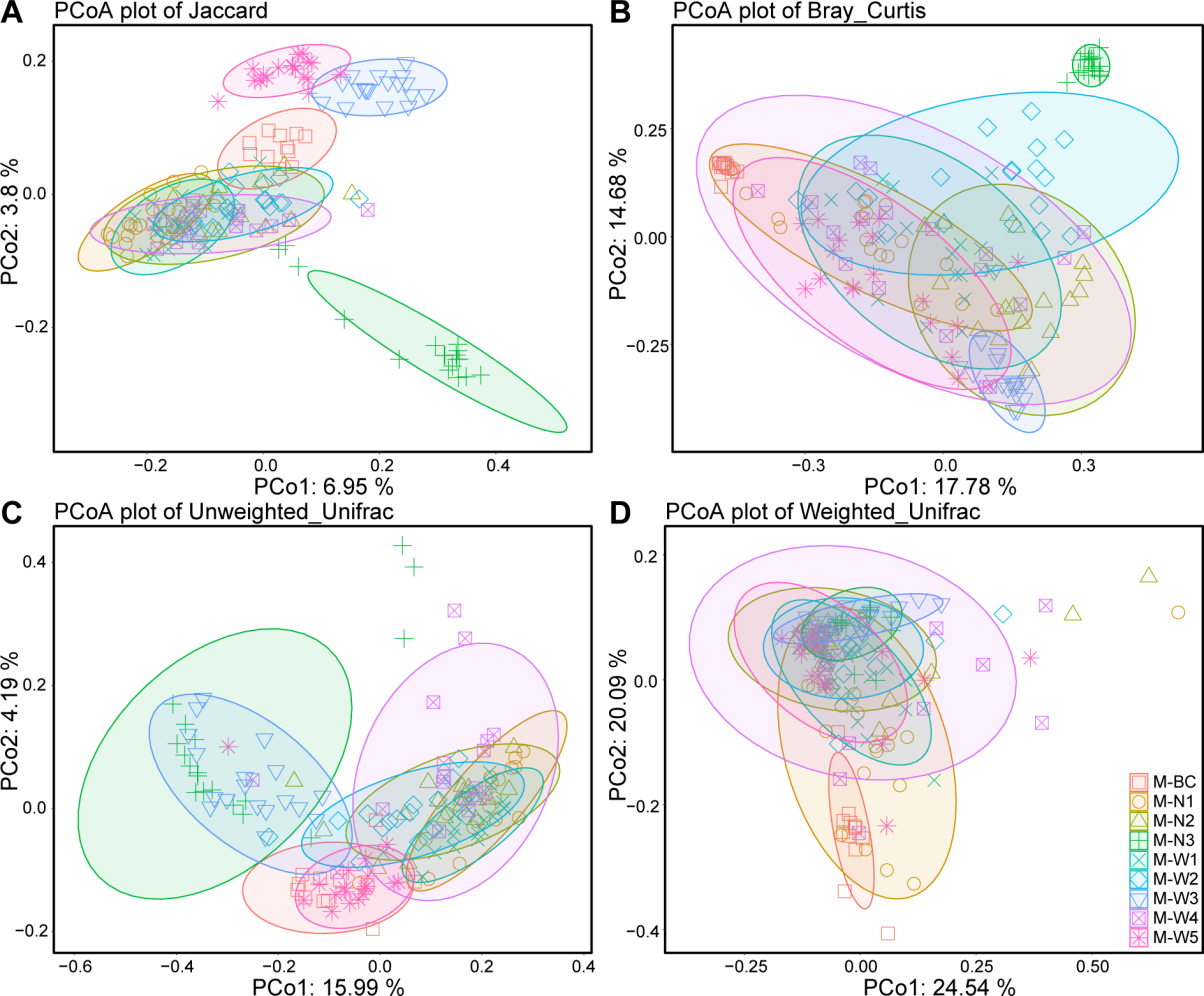
PCoA of Beta Diversity of microbial communities in lung tissues of drowned mice from eight sampling locations. (**A**) PCoA based on Jaccard distance. (**B**) PCoA based on Bray_Curtis distance. (**C**) PCoA based on Unweighted_Unifrac distance. (**D**) PCoA based on Weighted_Unifrac distance.

The microbial beta diversity in lung tissues of drowned mice in Day 1 groups from different water sampling points was analyzed using PCoA based on Jaccard, Bray_Curtis, Unweighted_Unifrac, and Weighted_Unifrac distances. The results showed that microbial beta diversity in lung tissues of drowned mice from eight water sampling points could not be significantly distinguished (Fig. 5). Among them, the M-N3 group could be distinguished independently from the other seven groups in both Jaccard and Bray_Curtis distance calculations, while the M-W3 group and M-W5 group could be distinguished independently from the remaining seven groups only in the Jaccard distance calculation. The remaining groups could not be independently clustered in any of the four distance calculations. These results underscore the impact of water quality parameters on microbial diversity within the Northwest River, where salinity and conductivity positively influenced microbial richness, while total phosphorus had a negative impact on microbial richness.

### Impact of submersion time on inference of drowning site

**Fig 6:**
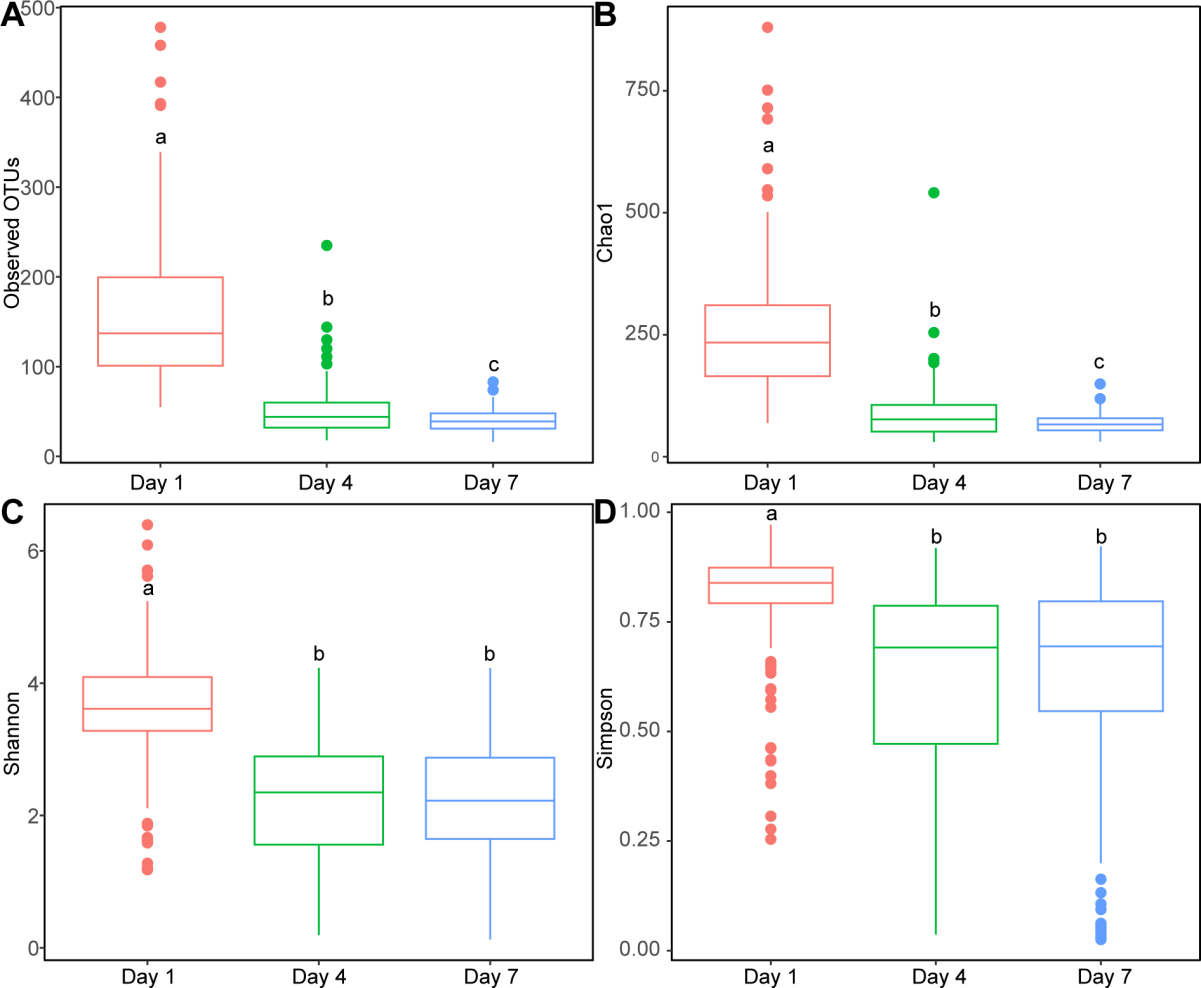
Alpha diversity indices of microbial communities in lung tissues of drowned mice with different drowning time intervals (A: Observed OTU numbers; B: Chao1 index; C: Shannon index; D: Simpson index. Different letters indicate statistical differences (*P* < 0.05)).

To investigate the impact of the submersion time on the inference of the drowning site, 16S rDNA sequencing was performed on lung tissue samples of mice drowned in water samples from different sampling points in the Northwest River after 1 day (Day 1 group), 4 days (Day 4 group), and 7 days (Day 7 group) after drowning. Alpha diversity analysis revealed that the Day 1 group exhibited significantly higher Observed OTUs, Chao1, Shannon, and Simpson indices compared to the Day 4 and Day 7 groups (Fig. 6). The Day 4 group showed a slight increase in Observed OTUs and Chao1 indices compared to the Day 7 group, but no significant differences were observed in Shannon and Simpson indices. These results indicate a significant influence of the submersion time on the Alpha diversity of microbial species in lung tissues, with a decrease in species richness and evenness with prolonged submersion time, particularly noticeable between Day 1 and Day 4. However, there was no significant change in microbial evenness from Day 4 to Day 7.

**Fig 7:**
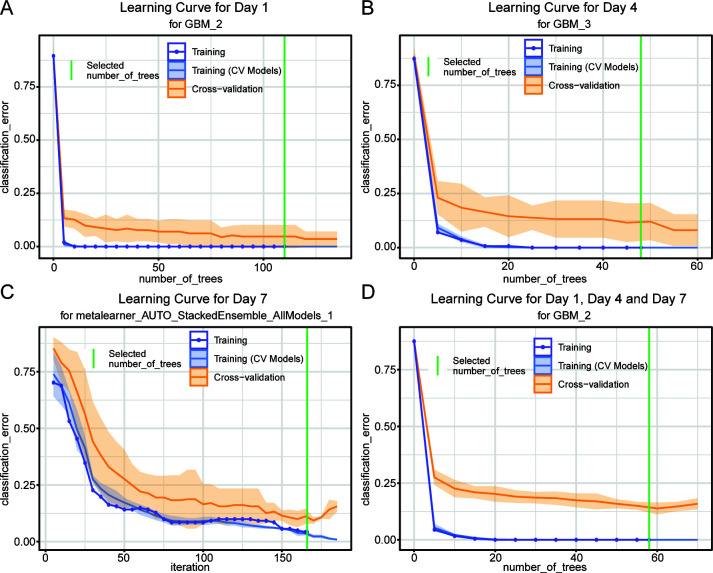
Learning curve of the GBM or metalearner model constructed by H2O automated machine learning algorithm using microbial community classification data from Day 1 (**A**), Day 4 (**B**), Day 7 (**C**), All Day 1, Day 4, and Day 7 (**D**). The red line indicates the selected number of trees.

To further validate the efficacy of machine learning in inferring drowning sites, we conducted machine learning modeling and validation using microbial classification data obtained from water samples collected at various time intervals following submersion in mouse lung tissues. The findings demonstrated that within each specific submersion time category, machine learning techniques effectively identified drowning sites (Fig. 7A through C ). The learning curves depicted in Fig. 7 illustrate the correlation between training error and validation error across different sizes of training data sets. These curves serve to evaluate the model’s performance and assess the degree of overfitting based on the fluctuation of errors as the training data size increases. The data set was partitioned randomly into five groups, with four groups allocated for modeling purposes and the remaining group for validation in a cross-validation setup. The cross-validation accuracy of the model was determined to be 95.07% ± 3.17% (Day 1 group, Fig. 7A), 87.60% ± 7.75% (Day 4 group, Fig. 7B), and 85.50% ± 7.55% (Day 7 group, Fig. 7C). When the microbial community classification data of Day 1, Day 4, and Day 7 groups were combined and modeled, the cross-validation results showed that the model can still achieve a classification accuracy of 83.53% ± 3.99% for different water sampling points (Fig. 7D). These results suggest that as the submersion time increases, the classification accuracy of the machine learning model gradually decreases, but still maintains at a relatively high level. In addition, the ability of models built using samples from specific submersion times to predict samples from other submersion times was also analyzed. The results (Fig. S6) showed that, when using data from the Day 1 group for modeling and testing on the Day 4 and Day 7 groups, the accuracies were 32.62% and 25.53%, respectively. When using data from the Day 4 group for modeling and testing on the Day 1 and Day 7 groups, the accuracies were 37.76% and 25.53%, respectively. Lastly, when using data from the Day 7 group for modeling and testing on the Day 1 and Day 4 groups, the accuracies were 18.18% and 56.03%, respectively. These findings underscore the importance of considering submersion time variations when applying machine learning models for drowning site inference.

### Impact of animal species on inference of drowning site

Drowning victims with different physical conditions may exhibit varying rates of decomposition in water due to differences in body tissues. This can affect the preservation status of the body and subsequently influence the inference of the drowning site. To investigate the impact of the drowning victim’s physical condition on the inference of the drowning site, drowning experiments were conducted in water samples from both the N1 and W5 sampling points using rabbits, aiming to analyze the influence of animal species on the inference of the drowning site compared to mice. The rabbits were grouped into the R-N1 and R-W5 groups. Alpha diversity analysis of rabbit lung tissue microbial 16S rDNA sequencing results showed no significant differences between the R-N1 and R-W5 groups in terms of Observed OTUs and Chao1 indices (Fig. S7A and B). However, the R-W5 group exhibited significantly higher Shannon and Simpson indices compared to the R-N1 group (Fig. S7C and D). Beta diversity analysis revealed that both the R-N1 and R-W5 groups could be distinctly separated in the PCoA plots generated from Jaccard, Bray_Curtis, Unweighted_Unifrac, and Weighted_Unifrac distance calculations (Fig. S8)

**Fig 8:**
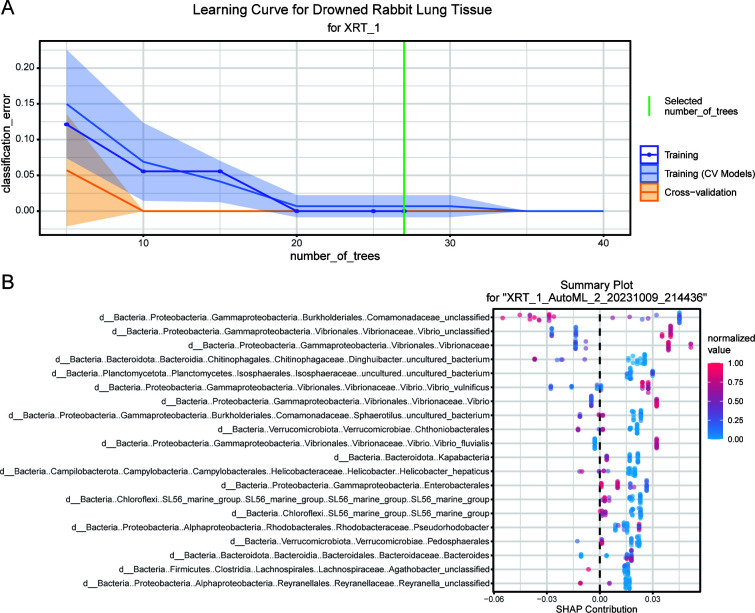
Learning curve model of drowned rabbit lung tissue. (**A**) The learning curve of the XRT model constructed by H2O automated machine learning algorithm using microbial community classification data from drowned rabbit lung tissue (red line indicates the selected number of trees). (**B**) Feature importance ranking based on SHAP values. The red portions in the feature values indicate higher values.

After modeling the microbial community data of the R-N1 and R-W5 groups together, the cross-validation results showed a classification accuracy of 100% for different sampling points (Fig. 8A). Using the SHAP (SHapley Additive exPlanation) tool for machine learning model interpretation, the plot indicated that the specie Comamonadaceae_unclassified had the highest importance, followed by the specie Vibrio_unclassified and the family Vibrionaceae (Fig. 8B).

When the machine learning model was trained using the Day 1 group data of drowned mice lung tissues and tested on the rabbit lung tissues of the R-N1 and R-W5 groups, the average accuracy was 61.11% (Table 1). Specifically, the prediction accuracy for the R-W5 group was 72.22%, while it was only 50.00% for the R-N1 group (Table 1). These results suggest that when inferring drowning sites across different animal species, relatively good accuracy can still be achieved, especially when there are significant differences between water samples from the target location and those from other locations.

**TABLE 1:** Statistics of drowning sites inference and accuracy for drowned rabbit lung tissue microbial data using multiclass machine learning classifier model GBM trained on drowned mouse lung tissue microbial abundance data from Day 1

Real value	Predicted value								Accuracy	Total accuracy
	N1	N2	N3	W1	W2	W3	W4	W5		
R-N1	9	0	0	2	1	5	1	0	50.00%	61.11%
R-W5	0	0	0	0	3	2	0	13	72.22%	

## DISCUSSION

Drowning incidents pose significant challenges for forensic investigators due to the complexity of determining the exact site where the drowning occurred. Traditional forensic methods often rely on physical evidence, witness statements, and circumstantial clues. However, the emerging field of forensic microbiology offers a promising avenue for enhancing the precision and reliability of drowning site inference. In this study, we investigated the microbial diversity in water samples and lung tissues of drowned animals from different sampling points in the Northwest River, to explore the potential of microbial signatures in inferring drowning sites.

Recent research has highlighted the utility of microbial analysis in forensic investigations, particularly in aquatic environments (26, 27). By analyzing the unique composition of microbial communities present in water samples and comparing them with those found in the lung tissues of drowned individuals, it is possible to establish a more accurate link between the victim and the site of drowning (28). This approach has shown promise in cases where traditional methods have yielded inconclusive results, providing valuable insights into the dynamics of microbial colonization post-submersion. Furthermore, advances in next-generation sequencing technologies have enabled a more comprehensive characterization of microbial communities, allowing for finer distinctions between different water bodies (29). By combining these molecular techniques with geographic information systems (GIS) mapping, investigators can create detailed profiles of microbial signatures associated with specific locations, aiding in the reconstruction of events leading to drowning incidents (30, 31). In our study, we collected water samples from multiple sites along the Northwest River, including the suspected drowning site and various control points upstream and downstream. In addition, lung tissues were analyzed to identify any shared microbial taxa between the water and the victim’s respiratory system. Our preliminary findings suggest a strong correlation between certain microbial species found in the water and those present in the lung tissues of the drowned victims, supporting the potential of microbial signatures as indicators of drowning sites.

The submersion time following drowning incidents plays a crucial role in the accuracy of site inference models based on microbial diversity. Alpha diversity analyses revealed a significant decrease in species richness and evenness over time. Samples collected 1 day after drowning (Day 1 group) exhibited higher Observed OTUs, Chao1, Shannon, and Simpson indices compared to samples collected at later time points (Day 4 and Day 7 groups). This decrease in alpha diversity suggests changes in microbial communities within lung tissues as a result of post-mortem processes and environmental factors. The observed temporal dynamics in alpha diversity align with previous studies on post-mortem microbial succession (32, 33). Miguel et al. demonstrated a rapid decrease in microbial diversity in decomposing pig carcasses, emphasizing the influence of time on microbial community dynamics (32). Similarly, Pechal et al. reported temporal shifts in bacterial communities during decomposition, highlighting the importance of considering post-mortem intervals in forensic microbial analyses (33). Machine learning classification using microbial data from different submersion times revealed variations in model performance. The cross-validation accuracy decreased with increasing submersion time elapsed since drowning. The Day 1 group achieved the highest classification accuracy, while the accuracy decreased for the Day 4 and Day 7 groups. This decrease in accuracy suggests that temporal changes in microbial compositions influence the effectiveness of the inference model. Temporal variability in model performance has been reported in various ecological studies (34–36). Ovaskainen et al. highlighted the importance of accounting for temporal autocorrelation in ecological modeling to improve predictive accuracy (34). The observed impact of time on the inference model highlights the importance of timely sample collection in forensic investigations of drowning incidents. Our findings suggest that microbial signatures in lung tissues undergo significant changes within the first few days following drowning, potentially compromising the accuracy of site inference models based on microbial diversity. Therefore, forensic investigators should prioritize the prompt collection of samples to enhance the reliability of site inference analyses.

Analysis of microbial community structures in lung tissue samples from drowned mice and drowned rabbits was conducted to simulate potential changes in microbial community structures in lung tissue post-drowning among different physiological populations. The results revealed that machine learning models developed using the microbial community abundance data from lung tissue samples of drowned mice (Day 1 group) could accurately infer the drowning sites of the Day 1 group, with an accuracy of 95.07% ± 3.17%. Similarly, machine learning models developed using microbial community abundance data from lung tissue samples of drowned rabbits could accurately infer the drowning sites of the rabbits, with an accuracy of 100%. However, when the machine learning model developed using microbial community abundance data from drowned mice (Day 1 group) was used to infer the drowning sites of drowned rabbits, the average accuracy was only 61.11%. Specifically, the prediction accuracy for the R-W5 group was 72.22%, while for the R-N1 group, it was only 50.00%. Song et al. demonstrated the importance of incorporating host phenotypic data, such as body weight, in predicting microbial community compositions in animal models, highlighting the relevance of host characteristics in microbial modeling (37). Furthermore, in beta diversity analysis of microbial communities in water samples from different drowning sites, the W5 group exhibited significant differentiation from the other groups, whereas the N1 group showed closer proximity to the W1 and W3 groups. These findings suggest that higher specificity in water microbial communities at drowning sites may compensate for the adverse effects of the drowned animal’s physiological characteristics on the inference of drowning sites.

While 16S rDNA sequencing provides useful taxonomic information at the genus or species level, whole-genome metagenomics would enable more detailed insights into the full microbial communities present, including strain-level differences, functional potential, and metabolic pathways that may be more directly related to the specific conditions of drowning and microbial degradation over time (38–40). This could further improve the accuracy and sensitivity of the model, especially in distinguishing between microbial communities associated with different aquatic environments or the time elapsed since drowning. For forensic applications, this could lead to enhanced resolution in estimating postmortem submersion intervals and distinguishing between geographic locations or environments of drowning cases. In addition, whole-genome metagenomics can reveal functional gene profiles, which may offer insights into environmental adaptations of microbial communities over immersion time.

In conclusion, the interdisciplinary approach harnessing microbiology to enhance forensic inference of drowning sites demonstrates significant potential in both cross-species animal inference and future applications in cadaver studies. Understanding the temporal dynamics and considering host characteristics are crucial for accurate site inference, contributing to case investigation and judicial trial. Future research endeavors should continue to explore and refine these models, ultimately advancing forensic science and facilitating justice.

## SUPPLEMENTAL MATERIAL

Supplemental figures and table spectrum.01321-24-s0001.docx Fig. S1 to S8; Table S1.

ASM does not own the copyrights to Supplemental Material that may be linked to, or accessed through, an article. The authors have granted ASM a non-exclusive, world-wide license to publish the Supplemental Material files. Please contact the corresponding author directly for reuse.
