# Supplementary material for: Integrating microbial profiling and machine learning for inference of drowning sites: a forensic investigation in the Northwest River
Source: Microbiol Spectr. 2024 Dec 9;13(1):e01321-24. doi: 10.1128/spectrum.01321-24 (PMC11705903; doi:10.1128/spectrum.01321-24)
Supplement: Supplemental figures and table — Fig. S1 to S8; Table S1. [file spectrum.01321-24-s0001.docx]

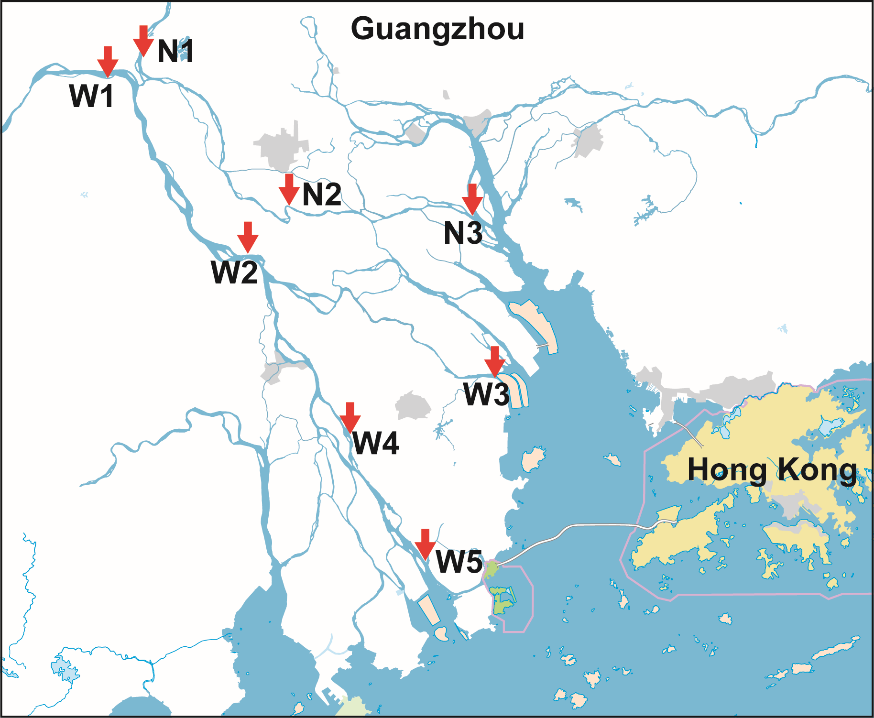


Fig. S1 Schematic Map of the Northwestern River Research Locations. Red dashed arrows indicate sampling points.


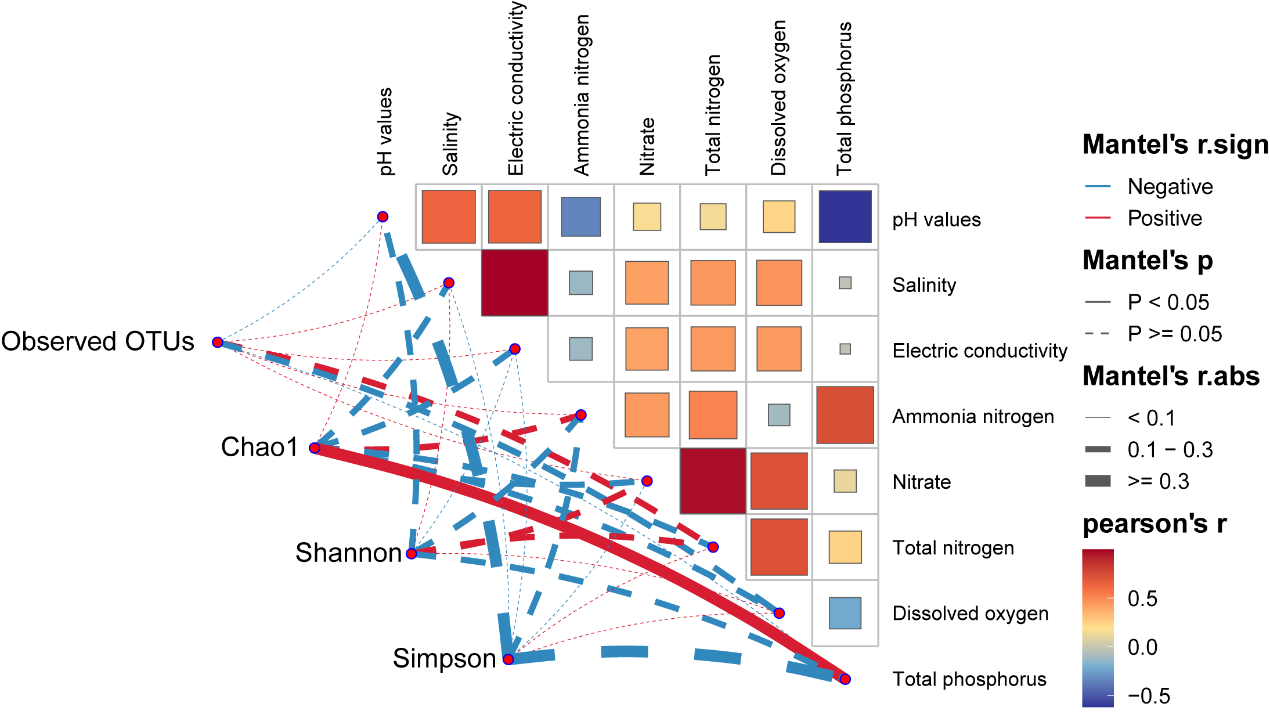


Fig. S2 Mantel correlation between Alpha diversity indices of microbial communities and water quality parameters at 8 sampling locations. Pearson correlation analysis was applied between water quality parameters. The width of connecting lines represents the magnitude of the correlation coefficient (Mantel’s r), while the color of the lines indicates the statistical significance based on Mantel’s *P*-values (solid lines for *P*<0.05, dashed lines for *P*≥0.05).


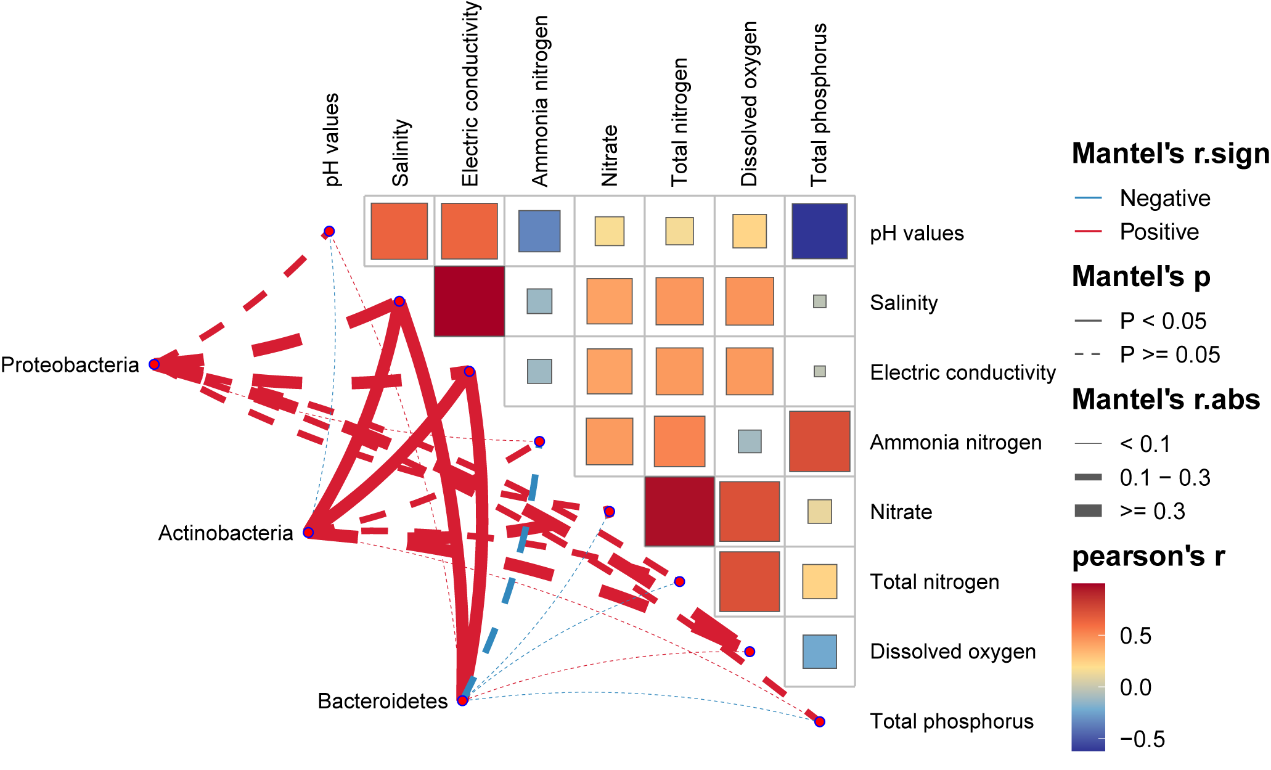


Fig. S3 Mantel correlation between dominant Phyla-level microbial communities (top 3) in water samples from 8 sampling locations. Pearson correlation analysis was applied between water quality parameters. The width of connecting lines represents the magnitude of the correlation coefficient (Mantel’s r), while the color of the lines indicates the statistical significance based on Mantel’s *P*-values (solid lines for *P*<0.05, dashed lines for *P*≥0.05).


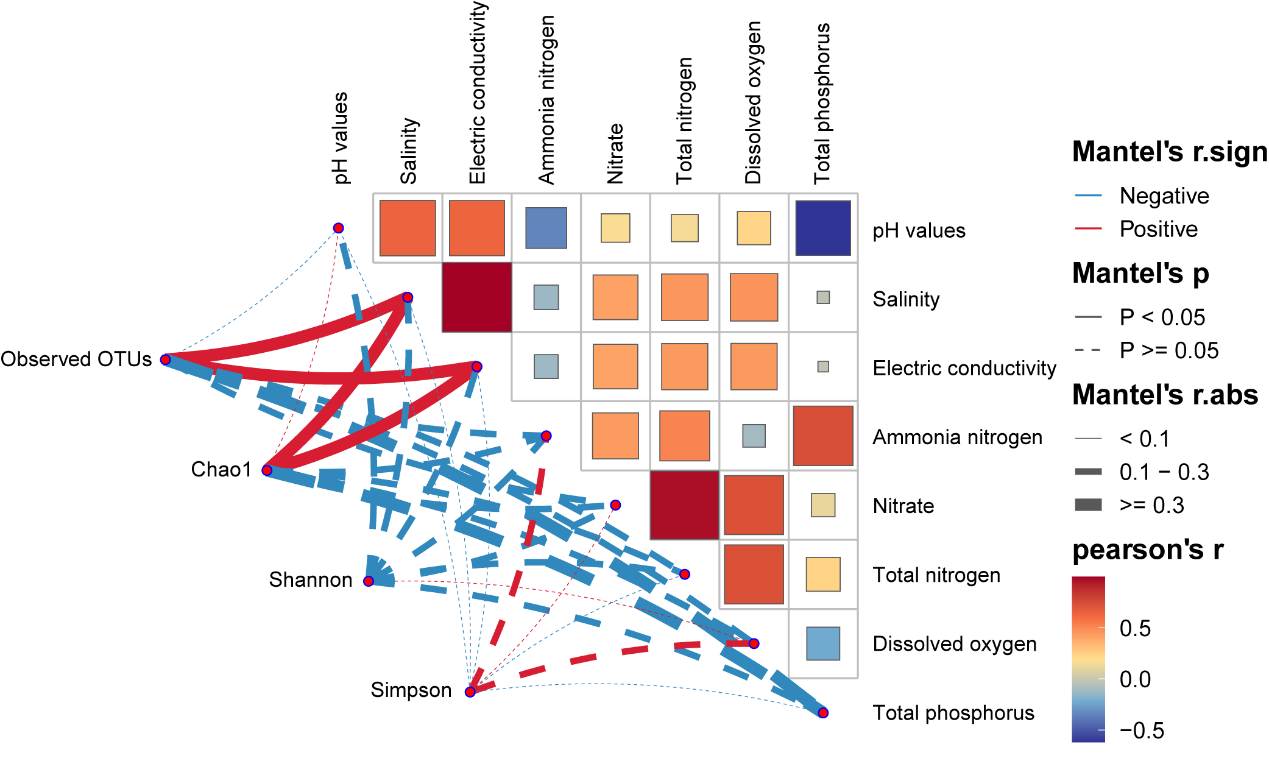


Fig. S4 Mantel correlation between Alpha diversity indices of microbial communities in lung tissues of drowned mice and water quality parameters at 8 sampling locations. Pearson correlation analysis was applied between water quality parameters. The width of connecting lines represents the magnitude of the correlation coefficient (Mantel’s r), while the color of the lines indicates the statistical significance based on Mantel’s *P*-values (solid lines for *P*<0.05, dashed lines for *P*≥0.05).


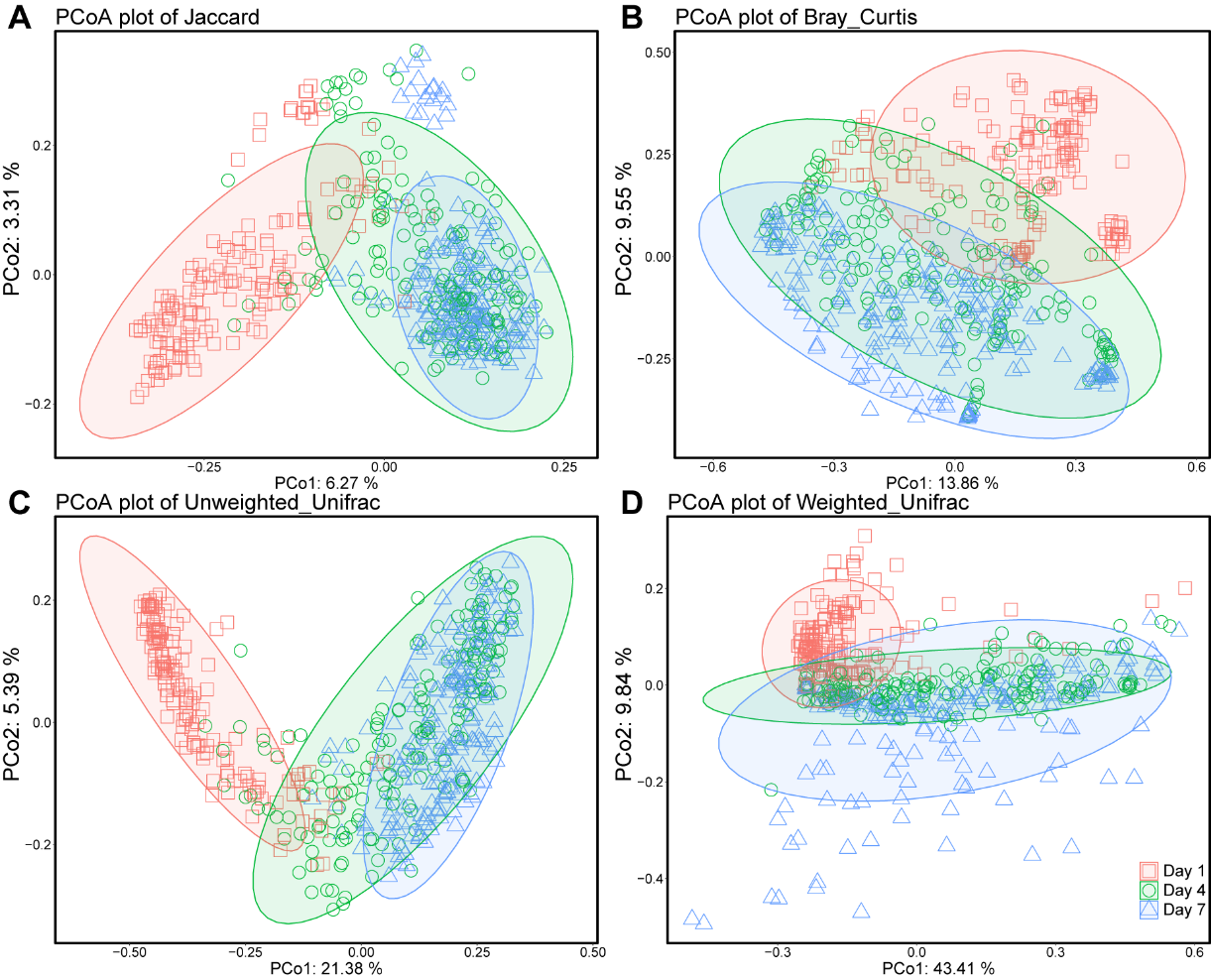


Fig. S5 Principal Coordinate Analysis (PCoA) of Beta Diversity of microbial communities in lung tissues of drowned mice with different drowning time intervals.. (A) PCoA based on Jaccard distance. (B) PCoA based on Bray_Curtis distance. (C) PCoA based on Unweighted_Unifrac distance. (D) PCoA based on Weighted_Unifrac distance.


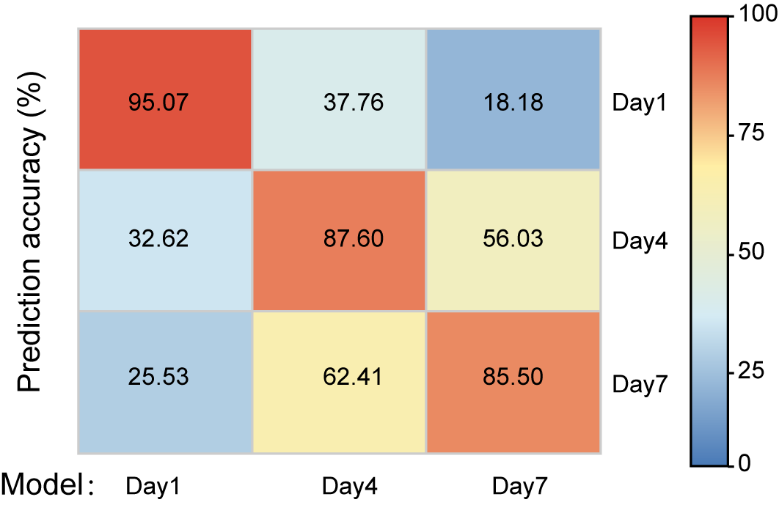


Fig. S6 Statistics of accuracy in predictions for specific submersion times’ data using the machine learning model trained on microbial abundance data of Day 1, Day4 or Day7.


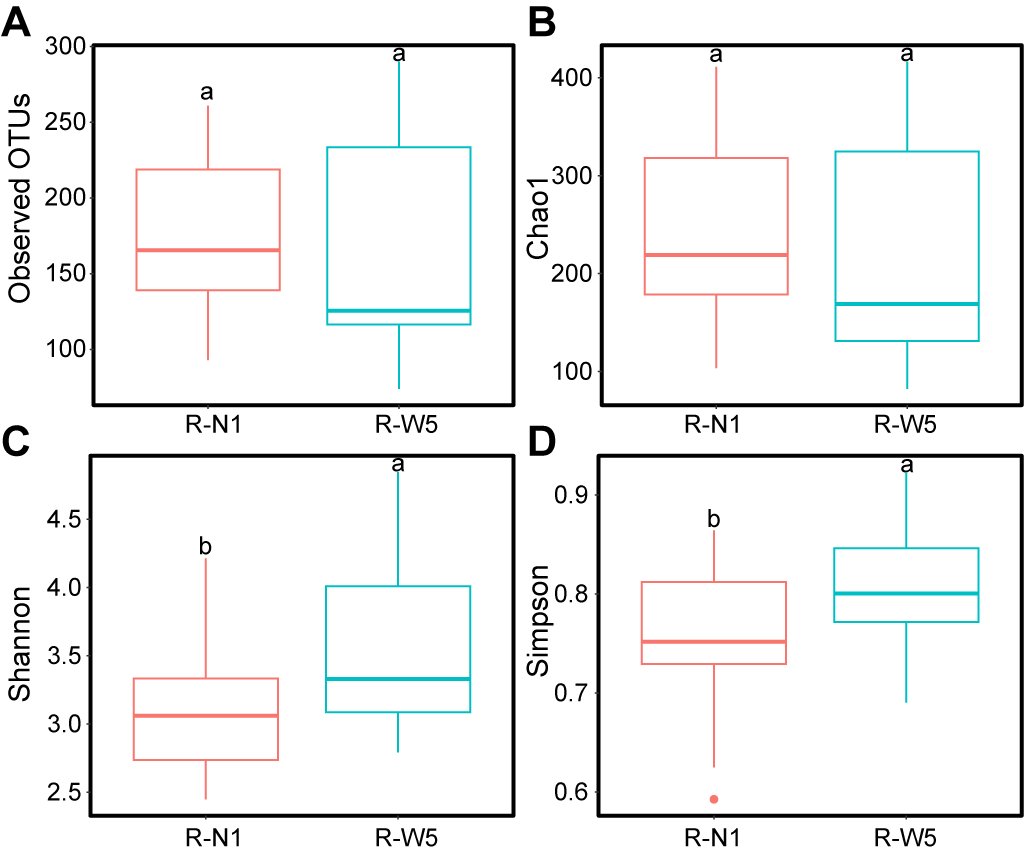


Fig. S7 Alpha diversity indices of microbial communities in lung tissues of drowned rabbits from 2 sampling locations (A: Observed OTU numbers; B: Chao1 index; C: Shannon index; D: Simpson index. Different letters indicate statistical differences (*P*<0.05)).


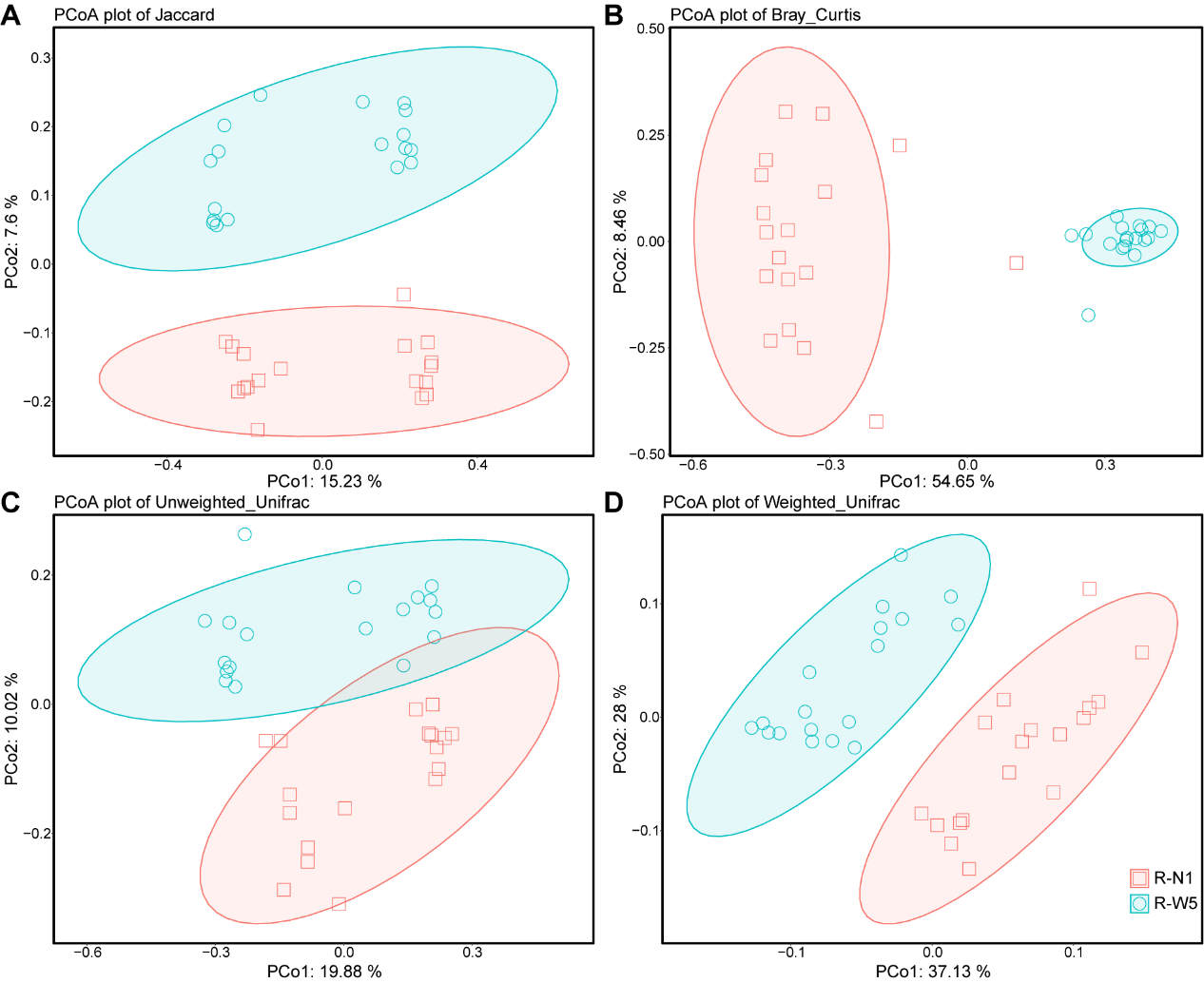


Fig. S8 Principal coordinate analysis (PCoA) of Beta diversity of microbial communities in lung tissues of drowned rabbits from 2 sampling locations. (A) PCoA based on Jaccard distance. (B) PCoA based on Bray_Curtis distance. (C) PCoA based on Unweighted_Unifrac distance. (D) PCoA based on Weighted_Unifrac distance.

Table S1 Water quality measurement results of samples from 8 sampling locations

| **Sampling Location** | **pH values** | **Salinity/ppt** | **Electric conductivity/us** | **Ammonia nitrogen mg/L** | **Nitrate mg/L** | **Total nitrogen mg/L** | **Dissolved oxygen mg/L** | **Total phosphorus mg/L** |
| --- | --- | --- | --- | --- | --- | --- | --- | --- |
| **N1** | 6.23 | 127.33 | 313.00 | 0.19 | 1.66 | 1.80 | 7.20 | 0.07 |
| **N2** | 6.17 | 125.00 | 283.67 | 0.18 | 1.64 | 1.94 | 9.27 | 0.08 |
| **N3** | 6.10 | 1.53 | 3.13 | 0.38 | 1.25 | 1.48 | 5.00 | 0.09 |
| **W1** | 6.60 | 110.33 | 254.33 | 0.02 | 1.56 | 1.73 | 8.03 | 0.03 |
| **W2** | 6.33 | 136.67 | 315.33 | 0.53 | 2.28 | 2.45 | 9.17 | 0.08 |
| **W3** | 6.10 | 3.09 | 4.82 | 0.24 | 1.86 | 2.02 | 8.40 | 0.07 |
| **W4** | 6.34 | 129.00 | 291.50 | 0.02 | 1.69 | 1.78 | 8.10 | 0.06 |
| **W5** | 6.08 | 5.66 | 11.16 | 0.14 | 1.59 | 1.66 | 8.20 | 0.05 |
